# Wound of the Antrum of Highmore, with Destruction of the Eye; Fragment of Knife-blade Removed from the Antrum Two Years Afterwards

**Published:** 1842-09

**Authors:** W. H. Donne

**Affiliations:** Louisville


Art. III.—*Wound of the Antrum of Highmore, with destruction of the eye; fragment of knife-blade removed from the antrum two years afterwards.* By W. H. Donne, M. D., of Louisville. Reported by R. S. Wendel, Student of Medicine.

Schuti, a gardener, aged 42 years, a native of Germany, in a rencontre with an athletic man, on the 3d of May, 1840, was struck with a dirk-knife which entered about an inch above the right superciliary arch, passed through the corresponding eyelid, downwards and backwards, evacuating the humors of the eye, and penetrated the antrum. The globe of the eye was divided by a vertical incision, through which the aqueous humor escaped; the iris was extensively detached at the ciliary margin, and could be partially seen through the transparent cornea—its surface being somewhat obscured by small coagula. The hemorrhage was slight, and easily controlled by moderate pressure. The patient complained of intense pain in the temple and cheek of the wounded side, shooting deep into the orbit. Three points of interrupted suture were used to approximate the edges of the divided eye. Lint saturated with laudanum and warm water, constituted the dressing.

May 4th.—Some tumefaction in the eyelid; pulse 110; tongue coated and dry; skin hot; patient has spent a very restless night. Ordered following medicine:

Tart. Emetic, gr. j.
Sulph. Magnesia, ℥ss.

to be dissolved in one-half pint of water, and a table-spoonful to be taken every half-hour, until nausea is induced—after which the interval may be increased.

May 5th.—Bowels freely evacuated; pain less; skin moist; pulse 90 and soft. From this period until the wound healed—the space of three weeks—no constitutional symptoms of an untoward character occurred. The patient, however, contended that a portion of the knife-blade remained in the roof of his mouth. But on the most careful examination no foreign body could be detected.

On the 10th of August, 1842, Mr. Schuti called and requested Dr. Donne to examine his mouth, stating that for six months past he had been annoyed by a rough projecting substance, which some person had informed him was a piece of dead bone, but which he believed to be the point of the knife, that had been driven down into the bone by the violence of the blow. On looking into the mouth a small black speck was discernible about one-half inch from the interval between the first and second molar teeth. The parts adjacent were somewhat tumefied and inflamed. Dr. Donne made several attempts to extract this body with a pair of common dissecting forceps, but found it immovably fixed in the substance of the bone. By dissecting around it with a bistoury down to the palate process of the superior maxillary bone, he was enabled to get a firmer hold, and with a pair of curved tooth forceps, succeeded in removing a fragment of the blade, 1¼ inches in length, and ¾ in. wide at the widest part; the extraction was not effected without considerable violence, and was attended with extreme suffering. The fragment came out with an audible snap which induced those present to suppose at first that it had been broken; but on inspecting its surfaces closely, they were found similarly oxidised and wanting the lustre which a recent fracture would have presented. Upon probing the aperture through which the fragment had been extracted, no other piece could be detected. This opening would scarcely admit the curved probe which Dr. Donne passed into the antrum, in order to satisfy himself that the whole of the foreign body was removed. The next day there was a slight discharge from the aperture, though the patient has suffered very little pain since the operation.

August, 1842.

[This case is certainly of a novel character. The rapidity with which the injury was repaired, and the length of time during which the fragment of the blade remained imbedded in the antrum, without causing much if any trouble to the patient, will at once strike the reader. It adds another to the many evidences on record of how much a sound constitution can effect, when aided by simple, yet prompt and judicious treatment such as was pursued by Dr. Donne in this instance. We should think it not improbable, however, that the patient may still have some difficulty, as the long sojourn of the broken knife in the antrum must have left the mucous membrane in a condition to take on disease from a slight cause. C.]

